# Local, collaborative, stepped, and personalized care management for older people with chronic diseases – results from the randomized controlled LoChro-trial

**DOI:** 10.1186/s12877-023-03797-2

**Published:** 2023-02-13

**Authors:** Gloria Metzner, Lukas Maximilian Horstmeier, Jürgen Bengel, Eva Maria Bitzer, Elena Dreher, Fabian Frank, Anne Göhner, Bernhard Heimbach, Ines Himmelsbach, Klaus Kaier, Jasmin Kiekert, Katharina Kohler, Katharina Laubner, Lisa Lyssenko, Andy Maun, Christoph Maurer, Claudia Salm, Jochen Seufert, Sebastian Voigt-Radloff, Erik Farin-Glattacker

**Affiliations:** 1 Section of Health Care Research and Rehabilitation Research, Institute of Medical Biometry and Statistics, Faculty of Medicine and Medical Center, University of Freiburg, Hugstetter Str. 49, 79106 Freiburg, Germany; 2 Department of Rehabilitation Psychology and Psychotherapy, University of Freiburg, Engelbergerstr. 41, 79085 Freiburg, Germany; 3 University of Education Freiburg, Public Health and Health Education, Kunzenweg 21, 79117 Freiburg, Germany; 4 Center for Geriatric Medicine and Gerontology, University Medical Center Freiburg, Faculty of Medicine and Medical Center, University of Freiburg, Lehener Str. 88, 79106 Freiburg, Germany; 5 Department of Social Work, Protestant University of Applied Sciences Freiburg, Bugginger Straße 38, 79114 Freiburg, Germany; 6 Department of Psychiatry and Psychotherapy, Medical Center - University of Freiburg, Faculty of Medicine, University of Freiburg, Hauptstraße 5, 79104 Freiburg, Germany; 7 Catholic University of Applied Sciences Freiburg, Karlstraße 63, 79104 Freiburg, Germany; 8 Institute of Medical Biometry and Statistics, Faculty of Medicine and Medical Center, University of Freiburg, Zinkmattenstr. 6a, 79108 Freiburg, Germany; 9 Division of Endocrinology and Diabetology, Department of Medicine II, University Medical Center Freiburg, Faculty of Medicine and Medical Center, University of Freiburg, Hugstetter Str. 55, 79106 Freiburg, Germany; 10 Institute of General Practice / Family Medicine, Medical Center – University of Freiburg, Faculty of Medicine, Elsässer Str. 2m, 79110 Freiburg, Germany

**Keywords:** Collaborative care, Care management, Intervention, Older adults, Chronic diseases, Multimorbidity, Evaluation, Randomized controlled trial

## Abstract

**Background:**

In the aging population of Western societies, an increasing number of older adults have multiple chronic diseases. As multifaceted health problems imply the involvement of several healthcare professionals, multimorbid older people frequently face a fragmentation of health care. Addressing these challenges, we developed a local, collaborative, stepped, and personalized care management approach (LoChro-Care) and evaluated its effectiveness.

**Methods:**

A two-group, parallel randomized controlled trial was conducted comparing LoChro-Care recipients (IG) to participants with usual care (CG). Patients aged 65 + with chronic conditions were recruited at inpatient and outpatient departments of the Medical Center, University of Freiburg. Participants were allocated using block randomization (n_IG_ = 261, n_CG_ = 263). LoChro-Care comprised individualized care provided by chronic care managers with 7 to 13 contacts over 12 months. Questionnaires were given at 3 time points (T_0_: baseline, T_1_: after 12 months, T_2_: after 18 months). The primary outcome was the physical, psychological, and social health status represented by a composite score of functional health and depressive symptoms. Secondary outcomes were the participants’ evaluation of their health care situation, health-related quality of life (HRQL), and life-satisfaction (LS). The data were analyzed using linear mixed modelling.

**Results:**

We analyzed *N* = 491 participants (n_IG_ = 244, n_CG_ = 247), aged M = 76.78 years (SD = 6.35). For the composite endpoint, neither a significant difference between IG and CG (*p* = .88) nor a group-time interaction (*p* = .52; *p* = .88) could be observed. Participants in both groups showed a significant decline on the primary outcome between T_0_ and T_2_ (*p* < .001). Post hoc analyses revealed a decline in both functional health (*p* < .001) and depressive symptoms (*p* = .02). Both groups did not differ in their evaluation of their health care situation (*p* = .93), HRQL (*p* = .44) or LS (*p* = .32). Relevant confounding variables were female gender and multimorbidity.

**Conclusion:**

Supporting patients’ self-management in coordinating their individual care network through LoChro-Care did not result in any significant effect on the primary and secondary outcomes. A decline of functional health and depressive symptoms was observed among all participants. Potential future intervention adaptations are discussed, such as a more active case management through direct referral to (in-)formal support, an earlier treatment initiation, and the consideration of specific sociodemographic factors in care management planning.

**Trial registration:**

German Clinical Trials Register (DRKS): DRKS00013904 (02.02.2018), https://drks.de/search/de/trial/DRKS00013904

**Supplementary Information:**

The online version contains supplementary material available at 10.1186/s12877-023-03797-2.

## Background

Enhancements in, for example, living conditions, nutrition, and medical treatments have led to increased life expectancy and, thus, to an aging population in Western societies. In Germany, the number of people aged 67 years and above increased by 54% between 1990 and 2018 – a trend that continues to rise [1]. With increasing age, the probability for the coexistence of several chronic health conditions, called multimorbidity, becomes more likely. A representative study in Germany showed that 75.8% of women and 68.0% of men aged 65–74 years have two or more coexisting chronic diseases, such as cardiovascular diseases, cancer, chronic pulmonary diseases, musculoskeletal disorders, diabetes mellitus, dementia, and depression [2]. Furthermore, the co-occurrence of multiple health conditions is associated with reduced functional capacity, loss of autonomy, poor self-reported health status, quality of life, need of help or even institutionalization, and mortality [2–6]. Considerably impaired functional health and poor perceived quality of life, in turn, were reportedly related with female gender, older age, being single or widowed, and low socioeconomic status [4, 5, 7]. In addition, research indicates a reciprocal association between impaired functional health, particularly physical impairments, and the onset and course of depressive symptoms [8, 9]. Hence, the patients’ functional health and depressive symptoms are considered to be highly relevant in this population.

Since multimorbidity is also associated with greater health care utilization [2, 3], this trend represents not only a major challenge for the health care system in general but also for individual health care provision. The presence of multimorbidity can thus lead to fragmented health care due to the involvement of numerous health professionals [10]. In this context, the World Health Organization (WHO) explicitly recommends a continuum of health care provision in terms of, i.e., coordinated, cross-sectoral care management [11]. Such interventions should aim at supporting preventive actions, improving functional ability, and averting or delaying adverse developments, rather than managing a single health condition in isolation [11].

However, most interventions have been developed and tested primarily with the focus on single diseases, such as depression, diabetes mellitus, and dementia [12–16], while studies evaluating complex care interventions for older adults with multiple chronic diseases are scarce. As shown by systematic reviews, only a few randomized controlled trials (RCT) have been carried out, none of which were conducted in Germany [17, 18]. Moreover, these studies showed mixed findings regarding the interventions’ effectiveness [17, 18]. In particular, studies either observed no improvements after the given treatment or results in favor of the control group on relevant clinical outcomes like functioning, cognition, quality of life, and depression [17].

In addition, literature reveals that information on the specific components of such care interventions is scarce, and limited knowledge about beneficial elements of complex care approaches for older multimorbid people exists [17, 19, 20]. Frequently identified components with potential impact are multidisciplinary teams, a comprehensive assessment, case management, care pathways/care plans, support for self-management, and education [11, 19–22]. As recommended in the German S3-treatment guideline for multimorbidity [6], the patient’s preferences, values, and needs should be prioritized. Therefore, elements such as shared decision-making and goal-setting also appear to be relevant components [11].

Given the literature described above, the development and evaluation of new approaches addressing the multiple needs and the resulting involvement of several health care providers in multimorbid older people is of great importance. In accordance with some of the aforementioned care elements and based on the “Ariadne principles” for patient-centered management of multimorbidity in primary care settings [23], we developed the LoChro-Care intervention – a new local, collaborative, stepped, and personalized care management approach for older people with chronic diseases (c.f. study protocol) [24]. It focuses on the enhancement of patients’ self-management in coordinating their individual care network in accordance with their health problems and subjective preferences. The objective of this study is to evaluate the effectiveness of LoChro-Care in terms of improvements in the physical, psychological, and social health status among older people with chronic diseases receiving LoChro-Care in comparison with usual care. We put forward the following hypotheses: (1) Older people receiving LoChro-Care will report an enhanced physical, psychological, and social health status as indicated by better functional health and reduced depressive symptoms. Moreover, LoChro-Care recipients will rate their (2) health care situation, as well as their (3) health-related quality of life and life-satisfaction, better than non-recipients.

## Methods

### Study design

In a two-group parallel RCT, LoChro-Care (intervention group, IG) was compared with usual care (control group, CG). The study was conducted at the Medical Center, University of Freiburg, Germany. Recruitment took place between January 2018 and March 2020. From the beginning of 2020, several procedures were adjusted due to the COVID-19 pandemic. From then on, we conducted the follow-up assessments, as well as the intervention contacts, exclusively by telephone. Ethical approval was granted by the ethics council of the University of Freiburg, Germany (495/17). The study follows the CONSORT guideline for reporting results from randomized trials [25] (see CONSORT Checklist – LoChro-study, additional file 1).

### Study recruitment

**Fig.1:**
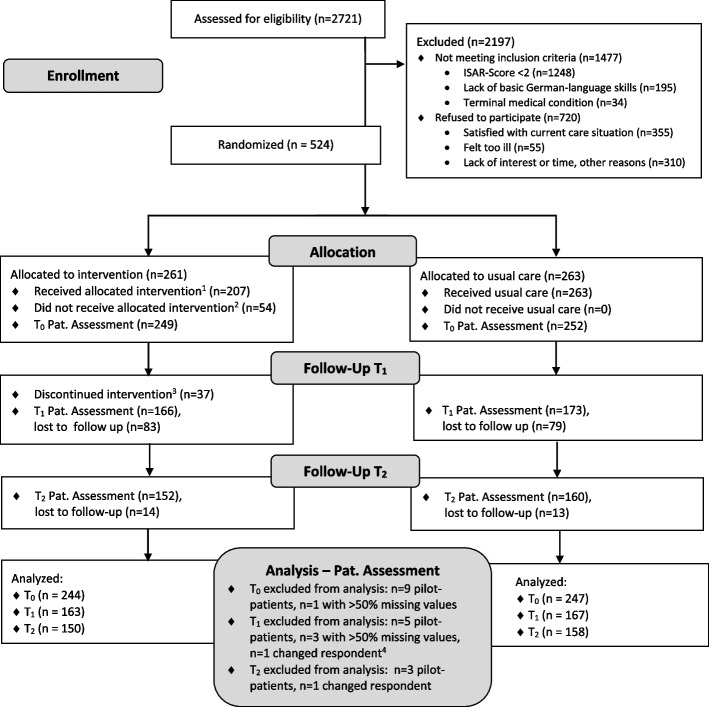
Flow diagram of the LoChro-study Note: ^1^Received intervention = participant received at least one session. ^2^Did not receive intervention = participant received no intervention due to the following reasons: death 27.78%, unable to be reached 12.96%, too much time expenditure 12.96%, decline of health condition 11.11%, no perceived need 11.11%, no longer interested 9.26%, relocation 5.56%, other reasons 9.26%. ^3^Discontinued intervention = participant discontinued intervention due to the following reasons: death 24.32%, unable to be reached 24.32%, decline of health condition 16.22%, no perceived need 8.11%, too much time expenditure 2.70%, no longer interested 2.70%, dissatisfaction with intervention 2.70%, relocation 2.70%, other reasons 16.22%. ^4^Changed respondent = in comparison to T_0_, the respondent changed from proxy to patient (while proxy assessments were generally excluded in the analysis reported here). Lost to follow up = reasons for lost to follow-up assessments were: death, unable to be reached, too much time expenditure, decline of health condition, no perceived need for intervention, no longer interested, relocation, dissatisfaction with intervention, changed respondent (proxy-assessment), or other reasons. ISAR = Identification of Seniors at Risk screening measurement (German Version; [26]). Pat. = Patient; Pat. Assessment = patients’ self-reported questionnaires

The a priori calculated sample size aimed for *n* = 606 study participants, assuming a dropout rate of 20% at the last follow-up time point (T_2_), a significance level of 0.05, 95% power, and a standardized mean difference between IG and CG of 0.30. Recruitment took place at the emergency center, selective wards, and at the geriatric, diabetes, and memory outpatient clinics of the Medical Center, University of Freiburg. Thus, we recruited both inpatients and outpatients, who were enrolled by research associates. Eligible patients were older adults aged 65 years or above with one or multiple chronical illnesses or geriatric symptoms who lived in Freiburg and nearby surrounding areas. In total, *n* = 2,721 potential participants took part in a short screening using the German version of the “Identification of Seniors at Risk” (ISAR) screening tool [26] (Fig. 1, flow diagram), which assesses the participants’ risk of unplanned readmission and need for nursing care. The inclusion criterion for study participation was an ISAR total score of two or more. Exclusion criteria were an ISAR score of less than two, a terminal medical condition, and insufficient German language skills. From the eligible patients, *n* = 1,477 did not meet the inclusion criteria and *n* = 720 refused to participate because they were satisfied with their current care situation or due to a lack of interest or time, for example (Fig. 1). Thus, the study contained *n* = 524 participants in total, from which *n*_IG_ = 261 were randomly allocated to the IG receiving the LoChro-Care, and *n*_CG_ = 263 CG-participants remained under usual care. We applied a block randomization without stratification. The allocation to the two groups was done by a research associate who was not involved in the recruitment, data collection procedure, or intervention provision using a computer-generated randomization schedule.

### Procedure

Patients, or the legal representative in the case of severe dementia or comparable conditions, granted written informed consent for study participation. Afterwards, the participants took part in the first questionnaire survey (baseline, T_0_). The follow-up questionnaire surveys took place 12 (T_1_) and 18 (T_2_) months later at the participants’ home (13.64% at T_1_, 5.86% at T_2_), if possible, or by telephone (86.36% at T_1_, 94.14% at T_2_). Responses were collected by research associates during individual interviews. In the case of medical conditions like dementia with limiting cognitive abilities for answering, the legal representative or relative responded to the questionnaire surveys (proxy-assessment). As our outcome variables primarily aimed at the subjective perception of health and psychosocial well-being, which can hardly be judged by third parties, these were not assessed via the proxy assessments. Thus, only the patients’ self-reported data are considered in the analysis reported here (patient assessment, see flow diagram Fig. 1).

### LoChro-Care intervention

**Fig. 2:**
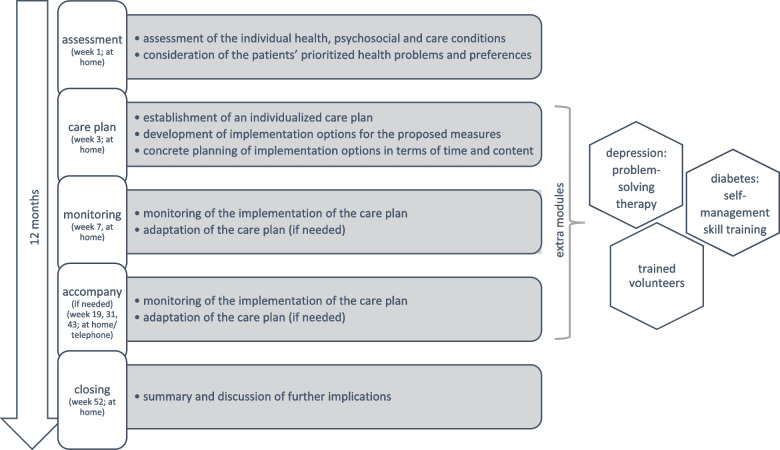
Elements of the LoChro-Care intervention

LoChro-Care was designed as a local, collaborative, stepped, and personalized care intervention. It focuses on enhancing the patients’ self-management in coordinating their individual care network by providing assistance to maintain or establish contact to formal and informal support (e.g., general practitioner, family, regional geriatric outpatient services). It comprised 7–16 contacts with trained chronic care managers (CCM) and lasted 12 months. A total of four CCM with extensive experience in the sectors of nursing, health education, and social work provided the intervention (qualifications: CCM 1: Bachelor of Science (BSc) Nursing Science, nurse; CCM 2: Master of Arts (MA) Health Education, nurse; CCM 3: MA Social Work; CCM 4: BSc Health Education, MA Social Work student). At least the first three contacts were home visits. Depending on the constitution of the patient, the monitoring contacts could also take place by telephone. From the beginning of 2020, the intervention contacts were conducted exclusively by telephone due to the COVID-19 pandemic. Figure 2 shows the elements of the LoChro-Care intervention. Following the “Ariadne principles” for the management of multimorbidity in primary care [23], LoChro-Care included: a) a comprehensive assessment of the individuals’ health, psychosocial, and care conditions; b) the development of an individualized care plan in accordance with the patients’ prioritized health problems and preferences; c) the implementation, adaptation, and monitoring of the care plan; and d) a closing session. Extra modules were provided for patients with mild depression or diabetes, which were administered by the CCM responsible for the respective patient. In the case of mild depression, six additional contacts were implemented, including a short problem-solving therapy. In the case of diabetes, three extra contacts were included that aimed at the improvement of the patients’ self-management skills concerning diabetes. Additionally, there was an option to involve trained volunteers for support if patients could not implement the care plan on their own and no primary caregiver was available. For the implementation of the intervention, standardized action plans for each intervention contact between the CCM and the patient were developed. In addition, standardized care plans for recurring patient issues were created (incl. informational materials) and adapted to the individual patients’ care situation, considering formal and informal support. Given the case of sufficient contact with the general practitioner and family caregivers, the CCM informed the patient about self-management and local geriatric outpatient services suitable for the patient’s needs. In the case of insufficient support, the CCM assisted with establishing contact with formal and informal care providers, e.g., the general practitioner, primary caregivers, trained volunteers, or geriatric services. In the monitoring sessions, the CCM and the patient collaboratively evaluated the implementation of planned actions, the patient’s current health problems, needs, and preferences, and adapted the care plan accordingly. The CCM themselves neither implemented any specific treatment measures nor connected the patients directly to health or psychosocial services. Instead, they identified appropriate care providers and assisted the patients with contacting them. In order to ensure treatment fidelity, the CCM had regular supervision sessions by an interdisciplinary geriatric team and research associates responsible for the process evaluation, as well as team intervision.

### Measurements

The same measurements were collected in each group at each time point. The primary outcome was the patients’ physical, psychological, and social health status. This was represented by a composite score consisting of the participants’ functional health and depressive symptoms, as these two impairments are assumed to be highly prevalent in the target population and reciprocally associated [8, 9]. The first component, functional health, was assessed using the “WHO Disability Assessment Schedule 2.0” (WHODAS 2.0) [27]; the latter, depressive symptoms, was assessed with the “Patient Health Questionnaire” (PHQ-9) [28]. The WHODAS assesses aspects of functional health during the last 30 days via six domains: cognition, mobility, self-care, interactions with other people, activities of daily living, and social participation. For an overall WHODAS-score, responses of the 32 items were counted, and the resulting score was transformed to a scale range of 0–100. Higher scores thereby indicate lower levels of functional health. The PHQ-9 measures depressive symptoms according to the DSM-IV criteria (Diagnostic and Statistical Manual of Mental Disorders) and consists of nine items (reference period: the last two weeks). The summary score ranges from 0–27, with a higher score indicating more severe depressive symptoms. In order to bring these differently measured outcomes to the same metric and, thus, to calculate the composite score, the Proportion-of-Maximum-Scaling (POMS) method was used [29]. Therein, each scale was transformed into a scale ranging from 0 (minimum) to 100 (maximum) using the following formula:

$$[(\mathrm{observed}\;\mathrm{value}\;-\;\mathrm{scale}\;\mathrm{minimum})\;/\;(\mathrm{scale}\;\mathrm{maximum}\;-\;\mathrm{scale}\;\mathrm{minimum})]\;\ast\;100$$

One secondary outcome was the patient’s evaluation of their health care situation. This was assessed by the “Patient Assessment of Chronic Illness Care” (PACIC) [30] (German version), which asks for the patients’ experiences with the described care elements within the last 6 months (11 items) and their overall satisfaction with health care (one item). For the analyses presented here, we used the one item of interest, namely: “In what percentage of cases was I satisfied with the organization of my medical care”. The two other secondary outcomes were health-related quality of life (HRQL) (using a self-developed item) and life-satisfaction (LS) [31]. Each of these was assessed with a single-item scale ranging from 0–10, with higher values indicating a higher HRQL or LS, respectively.

Multimorbidity was assessed using a weighted multimorbidity index, which can have values from zero to 37 [32]. Information on multimorbidity stemmed from the hospital’s documentation system and additional patient-reported diagnoses.

### Data analysis

All statistical analyses were executed using SPSS (version 28). Both intention-to-treat (ITT) and per-protocol (PP) analysis were performed. The statistician was blinded to the group allocation. An alpha-level of 0.05 (two-sided) indicated statistical significance.

The analysis compared change over time within the outcome variables between both groups. Linear mixed modelling (LMM) analysis was applied due to its robustness in handling missing data, as well as being able to handle both within- and between-subjects effects [33]. Intra-individual change over time was modelled on the first LMM level, while the subject-related inter-individual comparison was modelled on the second level [34]. For each primary and secondary outcome, a LMM was built following three consecutive steps: first, a random-intercept model (M_1_) for inter-class correlation (ICC) estimations was constructed including a random intercept for each participant. Second, a loaded model (M_2_), including all of the fixed effects with a respective choice of covariance matrix, was modelled. In a third step, the full model (M_3_) included the repeated measurement effect and defined covariance matrix [33]. The model fit was compared using likelihood ratio tests (LRT), based on the respective -2 log-likelihood indices and degrees of freedom per model [35].

Control variables were included since adjusted analyses are also recommend as sensible for RCTs when certain variables are expected to be prognostic [25]. Hence, the control variables comprised individual characteristics, namely *gender*, *age*, *degree of multimorbidity*, *family status*, *residential status*, *health insurance status*, and *educational level*, as well as self-reported *depression* and *diabetes* at baseline, as these two groups received specific LoChro-Care components. Additionally, differences in *recruitment path* (e.g., inpatient and outpatient setting) and *interview setting* (face-to-face vs. telephone) were included. The categorical control variables were dummy coded. The missing data and sample-dropouts were handled through the application of maximum likelihood estimation (restricted). As a long-format of the data set was used, the baseline values of each variable, as well as the values at T_1_ and T_2_, are represented through the combination with the measurement time point variable [33].

Systematic differences between dropouts and non-dropouts were examined with regards to baseline characteristics using t-tests for independent samples and contingency tables.

## Results

### Sample

At baseline, a total number of *N* = 501 participants were assessed (*n*_IG_ = 249 and *n*_CG_ = 252; flow diagram Fig. 1 “patient assessment”). The dropout rate was 32.34% (*n* = 162) after 12 months (T_1_) and 7.96% (*n* = 27) after 18 months (T_2_). Dropouts showed more impairments and depressive symptoms, and higher levels of multimorbidity. Unplanned inpatient hospitalizations (e.g., because of falls or stroke) were documented as Serious Adverse Events (SAE) in *n* = 28 cases, but we did not exclude these cases from our study. Exclusion criteria for the analysis were: a) pilot phase participation, b) more than 50% missing values, c) cases where the respondent changed from proxy to patient in comparison to baseline, and d) proxy assessment. The ITT sample consisted of *n*_ITT_ = 491 participants. IG-participants who did not finish the intervention with a closing session were excluded in the PP analysis. Among these excluded IG-participants, *n* = 54 (20.69%) did not receive LoChro-Care (e.g., because of death [*n* = 15, 27.78%] or decline in the health condition [*n* = 6, 11.11%]; Fig. 1). Additionally, *n* = 37 (14.18%) IG-participants discontinued the intervention. Thus, the PP sample consisted of *n*_PP_ = 408 cases.

At baseline, the mean age in the ITT sample was *M* = 76.78 (*SD* = 6.35) years, ranging from 65 to 94 years. In total, *n* = 275 (56.01%) participants were female, *n* = 229 (47.64%) were married, and *n* = 448 (91.75%) lived in their private homes. The multimorbidity-index mean was *M* = 5.46 (*SD* = 3.44), ranging from zero to 20. Further demographic characteristics of the ITT, as well as the PP sample, can be found in Table 1. The baseline means and standard deviations for all outcomes can be found in the supplementary Table S1 (additional file 2).

**Table 1:** Demographic baseline characteristics in the intention-to-treat (ITT; *N* = 491) and per-protocol (PP; *N* = 408) sample at the first measurement time point (T_0_)

	**Intention-To-Treat (ITT)**		**Per-Protocol (PP)**	
***Characteristic***	***M (SD)***	***N***	***M (SD)***	***N***
**Age**	76.78 (6.35)	491	76.68 (6.35)	408
**Multimorbidity**	5.46 (3.44)	489	5.46 (3.55)	408
	***n (%)***	***N***	***n (%)***	***N***
**Female**	275 (56.01%)	491	237 (58.09%)	408
**Depression**	63 (13.04%)	483	49 (12.22%)	408
**Diabetes Mellitus**	143 (29.18%)	490	121 (29.73%)	408
**Marital Status**				
Single	40 (8.15%)	491	30 (7.35%)	408
Divorced	72 (14.66%)	491	57 (13.97%)	408
Widowed	150 (30.55%)	491	124 (30.39%)	408
Married *(Reference Category)*	229 (47.64%)	491	197 (48.29%)	408
**Residential Status**				
Care Home	4 (0.81%)	491	3 (0.74%)	408
Senior Apartment	39 (7.94%)	491	32 (7.84%)	408
Private residence *(Reference Category)*	448 (91.75%)	491	373 (91.52%)	408
**Insurance**				
Assistance Insurance	71 (14.52%)	491	58 (14.25%)	408
Private Health Insurance	31 (6.34%)	491	28 (6.88%)	408
Statutory health insurance *(Reference Category)*	389 (79.14%)	491	322 (78.87%)	408
**Highest School Degree**				
Certificate of Secondary Education (CSE)	200 (40.82%)	480	161 (39.56%)	408
General Certificate of Secondary Education (GCSE)	140 (28.57%)	480	117 (28.75%)	408
No School Degree	13 (2.65%)	480	10 (2.46%)	408
High school degree/A-Levels *(Reference Category)*	127 (27.96%)	480	120 (29.33%)	408
**Recruitment**				
Ambulance	29 (5.91%)	491	27 (6.62%)	408
Flyer	133 (27.09%)	491	121 (29.65%)	408
Stationary recruitment *(Reference Category)*	329 (67.00%)	491	260 (63.73%)	408
**Phone Interview**	42 (8.63%)	491	37 (9.07%)	408

Reference Category refers to the respectively used variable category during dummy coding

### Preliminary analysis

For the composite score (ITT), the ICC with *r* = 0.54 was relatively high due to the nature of the repeated measurement design. The model specifications (loaded model and full model) significantly enhanced the model-fit (*Χ*_1_(21) = 438.61, *p* < 0.001; *Χ*_2_(2) = 6.36, *p* = 0.02) [36]. Further information on the model fit and likelihood-ratio tests for the LMM of each outcome can be found in Table S2 (additional file 3).

### Primary analysis

#### Primary outcome

In regards to the participants’ physical, psychological, and social health status, the final model (M_3_; ITT) indicates that the composite score was significantly affected by time (*b*_T0-T2_ = 7.88, *p* < 0.001; *b*_T1-T2_ = 4.53, *p* = 0.01), gender (*b* = 6.14, *p* < 0.001), depression (*b* = 6.99, *p* < 0.01), multimorbidity (*b* = 1.26, *p* < 0.001), private health insurance (*b* = 5.59, *p* = 0.02), and General Certificate of Secondary Education (GCSE) for the highest school degree (*b* = 4.09, *p* = 0.03). The effects of group (*b* = -0.24, *p* = 0.88) and group*time (*b*_T0-T2_ = -1.20, *p* = 0.52; *b*_T1-T2_ = -0.29, *p* = 0.88) were not significant predictors (see Table 2).

**Table 2:** Parameter estimates (absolute) of the linear mixed model (LMM; Model 3, REML estimation) for the primary and secondary outcome variables using both the intention-to-treat (ITT) and per-protocol (PP) versions of the data set

	**Composite Score**		**WHODAS**		**PHQ-9**		**PACIC**		**HRQL**		**LS**	
	*ITT*	*PP*	*ITT*	*PP*	*ITT*	*PP*	*ITT*	*PP*	*ITT*	*PP*	*ITT*	*PP*
***Fixed Parameters***												
Intercept	9.78	-1.59	1.93	-9.35	15.69	4.84	63.97	71.34**	5.16***	7.05***	3.91*	6.14**
Group												
Time [T0-T2]	7.88***	9.28***	11.04***	13.28***	4.82*	5.28*						
Time [T1-T2]	4.53*	4.85**	7.05***	7.72***								
Time*Group [T0-T2]												
Time*Group [T1-T2]												
Gender	6.14***	6.28***	6.54***	6.45***	5.58***	5.85***	-5.16** 5.20**		-.58***	-.63***	-.54*	-.58**
Age												
Mulitmorbidity	1.26***	1.34***	1.59***	1.64***	.91***	1.00***		-.61*	-.07**	-.07**		
Depression	6.99**	6.72**			9.98***	8.92***	-6.91**				1.15***	1.08***
Diabetes												
Family_1 (= single)									.62*		1.09**	.95*
Family_2 (= divorced)												
Family_3 (= widowed)												
Home_1 (= care home)												
Home_2 (= senior appartment)												
Insurance_1 (= assistance)												
Insurance_2 (= private)	5.59*	5.89*	7.03**	7.02*							-.68*	-.67*
School_1 (= CSE)												
School_2 (= GCSE)	4.09*	4.58*	6.03**	6.71**								
School_3 (= none)												
Recruitment_1 (= Ambulance)												
Recruitment_2 (= Flyer)							-7.75***	6.73***				
Setting												
***Random Effects***												
Individual Intercept	136.68***	134.41***	206.70***	201.31***	116.03***	110.28***	130.95***	129.72***	1.56***	1.44***	2.05***	2.07***
Repeated Measure	136.52***	133.28***	173.08***	167.44***	157.14***	154.53***	371.58***	361.56***	1.46*** 1.59*** 3.49***	1.48*** 1.63*** 3.38***	1.87*** 2.39*** 3.70***	1.80*** 2.49*** 3.83***

Note: M3 = full model (including all relevant specifications, Variance Components [VC] & Scaled Identity [ID] Matrix), the difference in display of the random repeated measure effect stems from different residual matrices chosen for the best fitting model (VC and Degrees of Freedom [DF], respectively), *ITT* = Intention-To-Treat, *PP* = Per-Protocol, *REML* = Residual Maximum Likelihood Estimation, *CSE* = Certificate of Secondary Education, *GCSE* = General Certificate of Secondary Education. ***p < .001; **p < .01; *p < .05. All non-significant parameters have been omitted for clarity reasons

Over time, the participants in both the IG and CG showed higher composite-score levels (*M*_T0_ = 28.44 [*SD* = 4.82]; *M*_T1_ = 32.83 [*SD* = 4.70]; *M*_T2_ = 35.72 [*SD* = 4.71]), indicating a general drop in their physical, psychological, and social health status (see Table 3). With regards to this decrease, the IG and CG did not differ significantly at any given time point (*F*_T0_[1, 727.81] = 0.02, *p* = 0.88; *F*_T1_[1, 914.36] = 0.08, *p* = 0.77; *F*_T2_[1, 932.58] = 0.60, *p* = 0.40).

**Table 3:** Pairwise comparison of the effects of group and time on the composite score as criterion based on the full linear mixed model (LMM, Model 3, REML estimation) using the intention-to-treat (ITT) version of the data set

**Group**		**M**	**SD**	**F(*df*)**	***p*-Value**	**Difference**
Intervention Group (IG)		31.96	4.80	.28 (*1, 439.94*)	.59	-.74
Control Group (CG)		32.70	4.65			.74
**Time**						
Time 0		28.44	4.82	10.20 (2*, 681.18*)	** < .001**	4.39 *(T*_*1*_*)*
						7.28 *(T*_*2*_*)*
Time 1		32.83	4.70			-4.39 *(T*_*0*_*)*
						2.90 *(T*_*2*_*)*
Time 2		35.72	4.71			-7.28 *(T*_*0*_*)*
						-2.90 *(T*_*1*_*)*
**Group** * **Time**						
Time 0	IG	28.32	4.97	.02 (*1, 727.81*)	.88	.24
	CG	28.56	4.80			-.24
Time 1	IG	32.56	4.87	.08 (*1, 914.36*)	.77	.53
	CG	33.09	4.72			-.53
Time 2	IG	35.00	4.86	.60 (*1, 932.58*)	.44	1.45
	CG	36.45	4.74			-1.45

Note: M_3_ full model (including all relevant specifications, Variance Components [VC] & Scaled Identity [ID] Matrix), *ITT* = Intention-To-Treat, *REML* = Residual Maximum Likelihood Estimation

Furthermore, the results in Table 2 indicate that female participants had significantly lower levels of physical, psychological, and social health status (*b* = 6.14, *p* < 0.001; female *M* = 34.45 [*SD* = 17.10]), male *M* = 28.33 [*SD* = 18.22]). Participants with lower degrees of multimorbidity displayed a significantly better health status (*b* = 1.26, *p* < 0.001; *r*[1071] = 0.30, *p* < 0.001) than those with higher levels of multimorbidity. Participants who entered this study with self-reported depression showed significantly lower levels of health status (*b* = 6.99, *p* < 0.01; *M*_DepYes_ = 42.09 [*SD* = 18.21]; *M*_DepNo_ = 29.98 [*SD* = 17.14]). This also applies to participants whose highest school degree was not GCSE-level (*b* = 4.09, *p* = 0.03; *M*_GCSEYES_ = 32.78 [*SD* = 18.08]; *M*_GCSENO_ = 29.63 [*SD* = 17.12]). Those who were not privately health insured indicated higher levels in the composite score (*b* = 5.59, *p* = 0.02; *M*_PrivateNo_ = 32.42 [*SD* = 17.86], *M*_PrivateYes_ = 21.92 [*SD* = 14.40]). These differences did not change over time and did not substantially differ between the ITT and PP samples (see Table 2).

#### Post-hoc analysis

Post-hoc LMM analysis with the WHODAS and PHQ-9, respectively, were executed to take a closer look at the decline in the participants’ health status in both groups over time. Time mainly affected a difference in the WHODAS scores (*b*_T0-T2_ = 11.04, *p* < 0.001; *b*_T1-T2_ = 7.05, *p* < 0.001), while the change in PHQ-9 scoring was much smaller (*b*_T0-T2_ = 4.82, *p* = 0.02; *b*_T1-T2_ = 2.49, *p* = 0.18). In each analysis, there was no significant difference between the IG and CG (*b*_WHODAS_ = -1.18, *p* = 0.52; *b*_PHQ-9_ = 0.70, p = 0.65), as well as no significant interaction between group and time (*b*_WHODAS_T0T2_ = 1.67, *p* = 0.42, *b*_WHODAS_T1T2_ = -0.79, *p* = 0.71; *b*_PHQ-9_T0T2_ = -2.39, *p* = 0.21, *b*_PHQ-9_T1T2_ = -1.38, *p* = 0.48; see Table 2). Furthermore, both outcomes were similarly and positively affected by gender (*b*_WHODAS_ = 6.54, *p* < 0.001; b_PHQ-9_ = 5.58, *p* < 0.001) and multimorbidity (*b*_WHODAS_ = 1.59, *p* < 0.001; b_PHQ-9_ = 0.91, *p* < 0.001), while only the PHQ-9 was significantly and positively affected by a self-reported diagnosis of depression at baseline (*b*_WHODAS_ = 3.83, *p* = 0.14; *b*_PHQ-9_ = 9.98, *p* < 0.001). Again, these results are comparable for ITT and PP.

#### Secondary outcomes

In regards to satisfaction with care, HRQL, and LS, none of these secondary outcomes were significantly affected by the intervention variables of group, time, or the interaction between group and time (see Table 2). In general, women showed significantly lower levels of satisfaction with care (*b* = -5.16, *p* < 0.01), HRQL (*b* = -0.58, *p* < 0.01), and LS (*b* = -0.54, *p* < 0.05). Levels of multimorbidity, a self-reported diagnosis of depression, family status, health insurance, and the recruitment path were significant predictors of only a few of these secondary outcomes.

## Discussion 

In this RCT, we analyzed the effectiveness of a newly developed local, collaborative, stepped, and personalized care management approach for older people with chronic diseases, LoChro-Care. The results revealed no significant differences between participants receiving the LoChro-Care intervention and participants with usual care on any of the primary or secondary outcome variables. Thus, no improvements in the participants’ physical, psychological, and social health status, as indicated by functional health and depressive symptoms, were observed. In addition, participants who received LoChro-Care did not rate their health care situation, HRQL, or LS better than participants with usual care did. In sum, LoChro-Care yielded no effect over and above usual care, although individualized support by a CCM was provided.

In contrast to previous interventions which only focused on single diseases [12–16], LoChro-Care explicitly aimed to address the multiple health problems older people can experience and the resulting involvement of several health care providers. On the part of the participants, we could infer that we have reached the target group. For example, as indicated by the mean WHODAS score that represents the degree of functional health impairments, our sample lay on the 90th percentile of the general population [27]. Thus, the sample can be classified as relatively highly burdened, although a great variance has been found that covers the two extremes of no impairments to great impairments. With regards to the intervention, LoChro-Care included several recommended care elements, such as a comprehensive assessment, individualized care plans, support for self-management, and education [11, 19, 21, 22]. More precisely, it focused on the enhancement of the patients’ self-management in coordinating their individual care network of formal and informal support. Even though LoChro-Care comprised the prioritization of the patients’ preference, collaborative decisions on the treatment plan and the focus on the patients’ care situation in accordance with the German treatment guideline for multimorbidity [6], we could not detect an intervention effect. One explanation might be that the functional impairments were too severe to be compensated by patient self-management support alone. Although the CCM developed individualized care plans, taking into account the patients’ constitution and context, and additional informal support by trained volunteers was offered, the implementation of the care plan may not have been actionable for some patients. Hence, the assistance of highly-burdened older people by a CCM might not only address patients’ self-management but also a more active case management through direct referral to formal and informal support, as well as treatments for specific health conditions. The CCM provided extra modules for depression and diabetes, but the extent and intensity of these modules may have been too small. The plausibility of these hypotheses could be explored by deeper analyses of our process evaluation data. Future research may evaluate the effectiveness of a modified LoChro-Care approach.

In addition, another potential explanation for the absence of an intervention effect might be that our study participants may have already started with a considerably high level of health problems, whose progression could no longer be delayed. Hence, LoChro-Care probably could not reveal an effect at all. In this regard, we observed a decline in functional health and increasing depressive symptoms across the study period in all participants. A period of 18 months can be a long time for older people, making degeneration and reduced functional capacity more likely. In that sense, a longitudinal study in older adults revealed that the decline in activities of daily living and gait speed – aspects of functional health – took place most rapidly [37]. Thus, it can be questioned whether care interventions that primarily aim at patients’ self-management have the potential to counteract functional decline, and if so, at what point in time an effective change in progression would still be possible. Hence, future interventions aiming at averting or delaying functional decline and disease progression should start early.

In line with the results reported here, previous RCTs that examined the effectiveness of interventions for older adults with multiple health complaints showed no clear superiority of the intervention group participants [17, 18]. Although the heterogeneity of the target population in these investigations and in our study was intentional, this may have also made it more difficult to demonstrate an effect. In this debate, it is criticized that previous RCTs used numerous different outcome measurements with partly unclear psychometric properties, making it difficult to interpret and compare the results [17]. In contrast and especially with regards to the primary outcome, we applied well-established and validated instruments in our study (WHODAS; [27], PHQ-9; [28]). Moreover, the questionnaires used could be considered as suitable for the assessment of relevant outcome variables commonly experienced in older people. For example, the WHODAS questionnaire asks for existing functional impairments in different areas, like restrictions in activities of daily living, self-care, and social participation, which may be reciprocally associated with depressive symptoms [8, 9]. In this context, future research could aim at the development of a core outcome set, which describes a consensus about central outcome variables relevant to the target population, integrating the experts’ and the patients’ perspectives. This could facilitate the evaluation of the effectiveness of an intervention and the comparability of the studies’ results.

### Limitations

Although we reached a comprehensive sample size in the context of geriatric research, which formed a sound basis for the statistical analyses, some limitations should be mentioned. Potential bias could result from the regional specificity and the exclusion criteria applied. The study area was restricted to Freiburg and surrounding areas. Specific characteristics of this area, like the relatively high socioeconomic performance, might have influenced the implementation of the intervention and study results. Therefore, future studies should investigate similar health care approaches for older people with multiple chronic diseases in other German areas for comparison. In addition, we did not include patients with terminal conditions or insufficient German language skills. These factors could likely be conditions occurring in the population of older multimorbid people and in ethnically diverse societies, which pose specific demands on the care management; therefore, they should be explored in future research.

Finally, the effect of the COVID-19 pandemic cannot be ruled out. From the beginning of 2020, several study procedures were adapted to the pandemic situation. The monitoring and closing sessions of the intervention were then primarily provided by telephone. In principle, this was not an issue of particular concern because the intervention design and manual had already included telephone contacts between the CCM and patients. Moreover, the telephone contacts were feasible in most cases. Nevertheless, the general negative effects of the COVID-19 pandemic, such as restricted contacts, reduced doctor visits, or impaired mood, could have interfered with the intervention and influenced the evaluation of its effectiveness.

### Practical implications

In sum, we can infer several practical implications for future research and practice as indicated above. Attempts to modify LoChro-Care or to develop new interventions for older multimorbid people could include more active assistance in establishing formal and informal supports. In addition, it could comprise comprehensive case management that goes beyond self-management support. In this regard, the patients’ degree of multimorbidity, severity of the already existing health conditions, and prognostic progression should always be considered, and the optimal time-point for treatment initiation needs to be determined. In addition, it would be worthwhile to give more attention to both specific diagnoses with potential impact on the health status (e.g., depression) and sociodemographic factors. In particular, gender-specific needs could be addressed in the provision of care. In this context, it could be beneficial to gain more insight into the patients’ individual needs and perceived helpful intervention components, as well as into the care managers’ perspective on feasible care elements. Therefore, future research could first use qualitative methods (e.g., interviews) to explore the demanded intervention elements. Such results, in turn, might inform the development of care interventions and facilitate the identification of potential effective care management elements in respect to the target group and intervention goal.

## Conclusion

In this study, we developed a new, local, collaborative, stepped, and personalized care management approach for older people with chronic diseases, LoChro-Care, which addressed the patients’ self-management in coordinating their individual care network. Notwithstanding, our results indicated no significant effect of LoChro-Care on any of the primary or secondary outcomes. In addition, the results revealed a decline in functional health and depressive symptoms over time in all participants. In view of the ongoing aging society, it could be worthwhile to adapt and evaluate supportive care interventions like LoChro-Care. These should target close patient support and specific sociodemographic and contextual factors in the population of interest, as well as an early implementation of the intervention to avert or delay the progression of health complaints and functional impairments.

## Supplementary Information

**Additional file 1.** CONSORT Checklist.

**Additional file 2:** **Table S1.** Descriptives of the LoChro primary and secondary outcomes in the intention-to-treat (ITT) and per-protocol (PP) sample at the first measurement time point (t0).

**Additional file 3: Table S2.** The inter-class correlation (ICC) values and likelihood ratio tests (LRT) of the differently specified linear mixed models (LMM) predicting the change in participants‘ composite score values over the period of three consecutive time points for both the intention-to-treat (ITT) and per-protocol (PP) versions of the data set (REML).

## Data Availability

The datasets generated and analyzed in the current study are not publicly available due to data protection reasons but are available from the corresponding author upon reasonable request.

## Abbreviations

BSc: Bachelor of Science
CCM: Chronic Care Manager
CG: Control Group
CONSORT: Consolidated Standards of Reporting Trials
DSM: Diagnostic and Statistical Manual of Mental Disorders
GCSE: General Certificate of Secondary Education
HRQL: Health-Related Quality of Life
ICC: Inter-Class-Correlation
IG: Intervention Group
ISAR: Identification of Seniors at Risk
ITT: Intention-To-Treat
LMM: Linear Mixed Modelling
LRT: Likelihood Ratio Tests
LS: Life-Satisfaction
MA: Master of Arts
PACIC: Patient Assessment of Chronic Illness Care
PHQ-9: Patient Health Questionnaire
POMS: Proportion of Maximum Scaling
PP: Per-Protocol
RCT: Randomized Controlled Trial
SAE: Serious Adverse Events
WHO: World Health Organization
WHODAS: World Health Organization Disability Assessment Schedule
