# Supplementary material for: Local, collaborative, stepped, and personalized care management for older people with chronic diseases – results from the randomized controlled LoChro-trial
Source: BMC Geriatr. 2023 Feb 13;23:92. doi: 10.1186/s12877-023-03797-2 (PMC9924193; doi:10.1186/s12877-023-03797-2)
Supplement: Supplementary file 2 — Additional file 2: Table S1. Descriptives of the LoChro primary and secondary outcomes in the intention-to-treat (ITT) and per-protocol (PP) sample at the first measurement time point (t0). [file 12877_2023_3797_MOESM2_ESM.docx]

Table S1. *Descriptives of the LoChro primary and secondary outcomes in the intention-to-treat (ITT) and per-protocol (PP) sample at the first measurement time point (t0)*

|  |  | **Intention-To-Treat (ITT)** | | |  | **Per-Protocol (PP)** | | |
| --- | --- | --- | --- | --- | --- | --- | --- | --- |
| ***Variable*** | ***Group*** | ***M*** | ***SD*** | ***n*** |  | ***M*** | ***SD*** | ***n*** |
| *Primary Outcomes* | |  |  |  |  |  |  |  |
| **Composite Score** |  | 30.3 | 17.8 | 470 |  | 29.8 | 17.7 | 388 |
|  | *IG* | 30.8 | 18.1 | 235 |  | 30.8 | 18.1 | 235 |
|  | *CG* | 29.9 | 17.6 | 235 |  | 28.4 | 16.9 | 153 |
| **WHODAS** |  | 33.5 | 20.9 | 474 |  | 33.0 | 20.8 | 392 |
|  | *IG* | 33.7 | 21.1 | 236 |  | 33.7 | 21.1 | 236 |
|  | *CG* | 33.4 | 20.8 | 238 |  | 31.9 | 20.4 | 156 |
| **PHQ** |  | 27.2 | 18.3 | 487 |  | 26.8 | 17.9 | 404 |
|  | *IG* | 27.8 | 18.4 | 246 |  | 27.8 | 18.4 | 246 |
|  | *CG* | 26.5 | 18.3 | 241 |  | 25.2 | 17.2 | 158 |
| *Secondary Outcomes* | |  |  |  |  |  |  |  |
|  |  |  |  |  |  |  |  |  |
| **HRQL** |  | 4.9 | 2.3 | 489 |  | 4.9 | 2.2 | 406 |
|  | IG | 4.8 | 2.2 | 247 |  | 4.8 | 2.2 | 247 |
|  | CG | 5.0 | 2.3 | 242 |  | 5.2 | 2.2 | 159 |
| **SL** |  | 6.4 | 2.5 | 487 |  | 6.4 | 2.5 | 404 |
|  | IG | 6.2 | 2.6 | 245 |  | 6.2 | 2.6 | 245 |
|  | CG | 6.6 | 2.4 | 242 |  | 6.8 | 2.3 | 159 |
| **PACIC** |  | 34.1 | 17.0 | 489 |  | 34.2 | 17.1 | 406 |
|  | IG | 34.3 | 16.7 | 247 |  | 34.3 | 16.7 | 247 |
|  | CG | 34.0 | 17.3 | 242 |  | 34.2 | 17.8 | 159 |
