# Supplementary material for: Local, collaborative, stepped, and personalized care management for older people with chronic diseases – results from the randomized controlled LoChro-trial
Source: BMC Geriatr. 2023 Feb 13;23:92. doi: 10.1186/s12877-023-03797-2 (PMC9924193; doi:10.1186/s12877-023-03797-2)
Supplement: Supplementary file 3 — Additional file 3: Table S2. The inter-class correlation (ICC) values and likelihood ratio tests (LRT) of the differently specified linear mixed models (LMM) predicting the change in participants‘ composite score values over the period of three consecutive time points for both the intention-to-treat (ITT) and per-protocol (PP) versions of the data set (REML). [file 12877_2023_3797_MOESM3_ESM.docx]

Table S2. *The inter-class correlation (ICC) values and likelihood ratio tests (LRT) of the differently specified linear mixed models (LMM) predicting the change in participants‘ composite score values over the period of three consecutive time points for both the intention-to-treat (ITT) and per-protocol (PP) versions of the data set (REML)*

|  | ***ITT*** | | | | ***PP*** | | | |
| --- | --- | --- | --- | --- | --- | --- | --- | --- |
|  |  |  | |  |  |  | |  |
| **ICC** | .54 |  | |  | .54 |  | |  |
|  |  |  | |  |  |  | |  |
| **Var(Pat)** | 174.09 | (16.69) | | p<.001 | 171.87 | (17.48) | | p<.001 |
| **Var(Error)** | 147.87 | (8.55) | | p< .001 | 147.11 | (8.76) | | p< .001 |
|  |  |  | |  |  |  | |  |
|  | *M_0_* | *M_1_* | | *M_2_* | *M_0_* | *M_1_* | | *M_2_* |
| **LRT** | F(21)= 438.61,p<.001 | | F(2)= 6.36, p= .02 | | F(21)= 419.64,p<.001 | | F(2)= 8.09, p< .01 | |
|  |  |  | |  |  |  | |  |
| **-2Log** | 9049.61 | 8611.00 | | 8604.64 | 8073.57 | 7653.93 | | 7645.84 |
| **AIC** | 9053.61 | 8615.00 | | 8608.65 | 8077.57 | 7657.93 | | 7649.84 |
| **BIC** | 9063.57 | 8624.77 | | 8618.52 | 8087.31 | 7667.58 | | 7659.48 |

*Note: M_1_= random intercept model (without the predictors; Scaled Identity [ID] Matrix), M_2_= loaded model (without the longitudinal effect; Variance Components [VC] Matrix), M_3_= full model (Including all relevant specifications; VC & ID Matrix); ITT= Intention-To-Treat, PP= Per-Protocol, ICC= Inter-Class Correlation, REML= Residual Maximum Likelihood Estimation, Var( )= Variance, LRT= Likelihood-Ratio Test, -2Log= -2 Log-Likelihood, AIC= Akaike Information Criterion, BIC= Bayes Information Criterion.*
